# Delivery and actuation of aerosolized microbots

**DOI:** 10.1002/nano.202100353

**Published:** 2022-03-07

**Authors:** Coy J. Zimmermann, Tyler Schraeder, Brandon Reynolds, Emily M. DeBoer, Keith B. Neeves, David W.M. Marr

**Affiliations:** 1 Department of Chemical and Biological Engineering, Colorado School of Mines, Golden, Colorado 80401, USA; 2 Department of Pediatrics, University of Colorado Denver Anschutz Medical Campus, Aurora, Colorado 80045, USA; 3 Departments of Bioengineering and Pediatrics, Hemophilia and Thrombosis Center, University of Colorado Denver Anschutz Medical Campus, Aurora, Colorado 80045, USA

**Keywords:** Aerosolization, colloids, microbots

## Abstract

For disease of the lung, the physical key to effective inhalation-based therapy is size; too large (10′s of μm) and the particles or droplets do not remain suspended in air to reach deep within the lungs, too small (subμm) and they are simply exhaled without deposition. μBots within this ideal low-μm size range however are challenging to fabricate and would lead to devices that lack the speed and power necessary for performing work throughout the pulmonary network. To uncouple size from structure and function, here we demonstrate an approach where individual building blocks are aerosolized and subsequently assembled in situ into μbots capable of translation, drug delivery, and mechanical work deep within lung mimics. With this strategy, a variety of pulmonary diseases previously difficult to treat may now be receptive to μbot-based therapies.

## 1 | INTRODUCTION

The promise of microscale devices capable of medical intervention has led to the development of microbots (μbots) that swim, crawl, and roll.^[1–4]^ With sizes ranging from the 10′s to 1000 μm^[5]^ and designed for movement and delivery through the blood stream or GI tract, potential applications range from disease diagnosis^[6]^ to targeted therapies for stroke^[7]^ and cancer.^[8]^ For diseases of the lung however, aerosolization provides a more direct route for delivery to the airway. Aerosol-based therapies have been used for centuries to treat asthma and persistent cough and, with the advent of metered dose inhalers in the 1950′s, use has significantly increased.^[9]^ The efficiency and effectiveness of aerosolized treatment however is significantly reduced in diseases where fluid buildup creates transport barriers to underlying biofilms and epithelial cells.^[10,11]^ Common examples include pneumonia, cystic fibrosis, acute bronchitis and chronic obstructive pulmonary disease. With their potential to enhance in vivo transport, μbots could be used to overcome fluid buildup and enhance treatment. Often fabricated using techniques adapted from the microelectronics industry,^[12]^ μbots can be powered and directed by a variety of fields, including magnetic,^[13]^ acoustic,^[14]^ chemical,^[15]^ and even optical fields.^[16]^ For in vivo application, μbots are most commonly controlled via magnetic fields which do not attenuate in tissue^[17]^ and have demonstrated directed translation via swimming^[18,19]^ and rolling^[7,20]^ for drug delivery^[2,21]^ within aqueous environments. Delivery through air for lung-based therapies however requires additional considerations that limit the use of most current μbot strategies. With physical principles similar to those for air borne transmission of disease,^[22]^ inhaled drugs must be formulated within a specified size range. The optimum aerodynamic size for drug-laden aerosols is in the range of 1–5 μm,^[23]^ commonly delivered via nebulizer to define a desired particle size distribution that determines the deposition profile within the lungs.^[24]^ Here, we aerosolize 4.5 μm building blocks via droplets that, once delivered into a liquid film within the lung, can subsequently assemble into larger μbots that can quickly translate at speeds up to 200 μm/s and perform work.

## 2 | RESULTS AND DISCUSSION

Viscosity plays a dominant role in locomotion at small length scales.^[25]^ Microorganisms overcome this through physical adaptations, like rotating flagellum, that are difficult to artificially replicate and control.^[26,27]^ In a particularly nonbiomimetic approach, we have demonstrated a rapid and reversible μbot fabrication and powering method where μm-scale superparamagnetic beads assemble into μwheels upon application of a rotating magnetic field.^[20]^ These μwheels roll rapidly and can be immediately redirected with a simple alteration in the magnetic field orientation resulting in speed and heading changes. Because the approach relies on the assembly and rotation of μbots in a weak magnetic field, it does not require high fields and strong field gradients necessary for magnetophoresis. We note that, because beads are available with a variety of surface functional groups, a variety of biological agents can be attached to the surface and the μwheels used as a drug delivery vehicle.^[7]^

**FIGURE 1:**
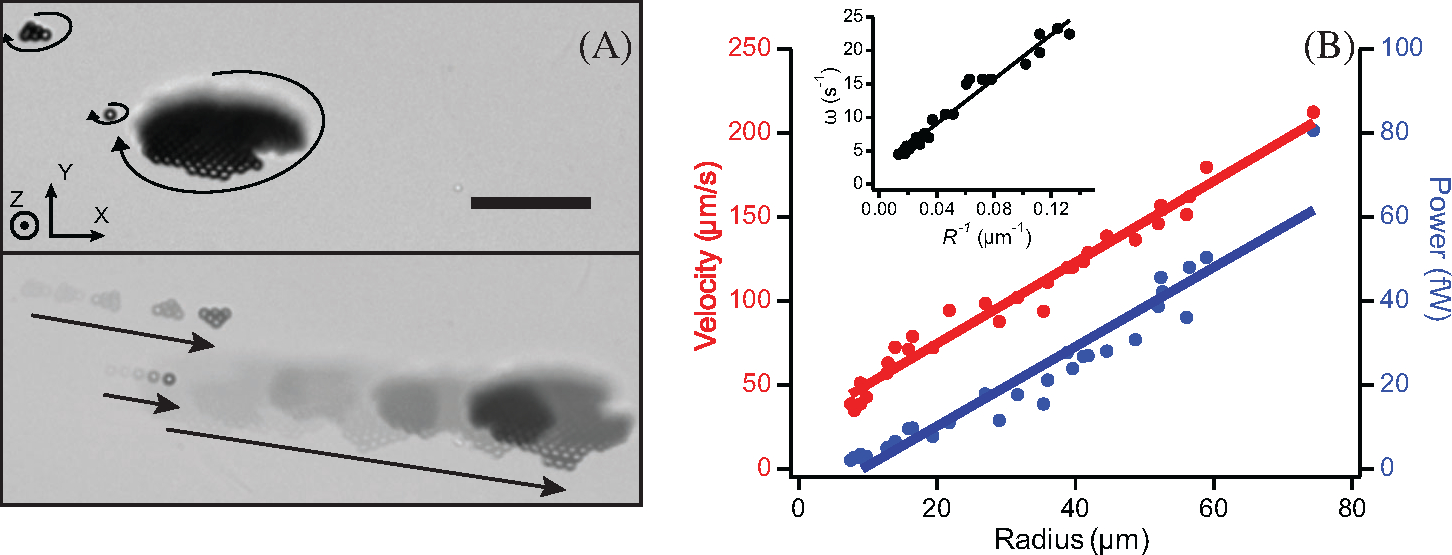
A, Wheel rotation and translation in 2 s with applied rotating magnetic field with axis of rotation $\overset{ˆ}{\Omega }=[0,\;cos(\pi /6),-1/2]$. 4.5 μm diameter beads, f=40Hz, magnetic flux density B = 3.4 mT, scale bar = 50 μm. Note larger μwheels translate faster than smaller ones (Supplementary Video 1). B, Velocity and power dependence on assembled μwheel size with linear fits to expected behaviors. Inset: μWheel rotation rate ω scales as 1/R

**FIGURE 2:**
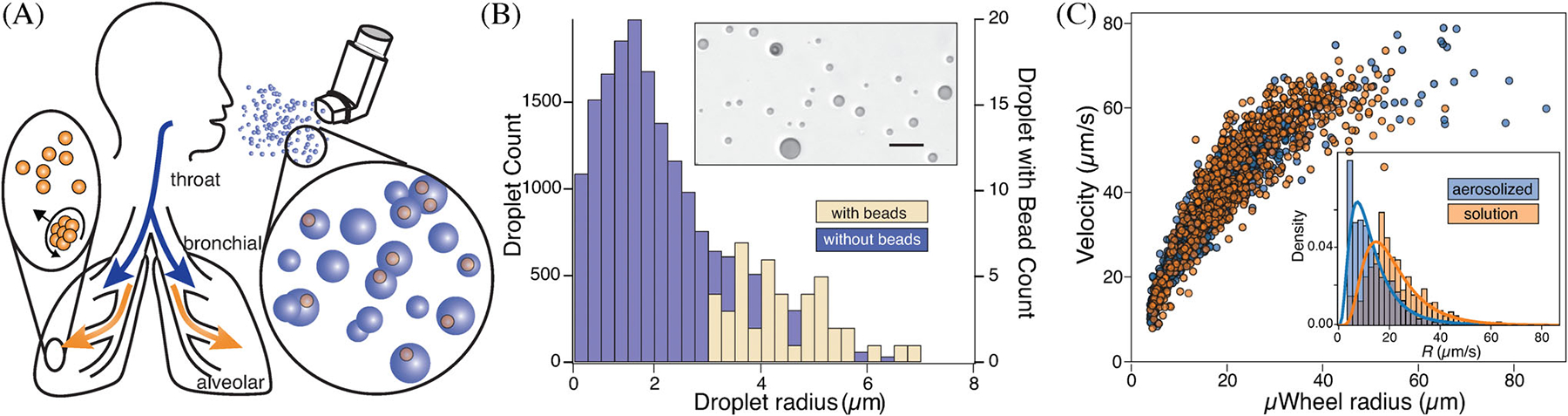
A, Concept illustration. B, Measured size distribution of aerosolized droplets with bead-containing drops identified. The overall fraction of droplets containing beads = 0.235%. Scale bar = 20 μm. C, Pre- and postaerosolization μwheel sizes and velocities f=40Hz, B = 2.1 mT. Note that both demonstrate similar behavior with size; however, a histogram of μwheel radii (inset) shows the μwheel distribution post aerosolization is shifted to smaller sizes

In this approach upon application of a magnetic field, superparamagnetic beads experience strong attractive interactions, bringing them together to assemble into two-dimensional structures of varying shapes and sizes. With rotation of the magnetic field, these structures spin and, with orientation of the field axis off the surface normal, μwheels translate (Figure 1A). Under fixed applied field conditions we measure the radius R, the rotation rate ω, and the translational velocity V to determine the power P via the rotational torque required to spin the μwheel. With μwheels powered via rotating magnetic fields of magnitude H, the magnetic torque induced can be expressed^[28]^ as

(1) $$\tau =N\nu \mu_{0}\chi^{\prime \prime }H^{2}$$

where N is the number of beads in the μ wheel, n the volume of an individual bead, $\mu_{o}$ the permittivity of free space, and $\chi^{\prime \prime }$ the imaginary part of the magnetic susceptibility. By approximating the viscous rotational μwheel torque with that of a disk^[29]^

(2) $$\tau =32\eta \omega R^{3}/3$$

with η the viscosity, one obtains

(3) $$\omega =3N\nu \mu_{o}\chi^{\prime \prime }H^{2}/32\eta R^{3}$$

and, with a μwheel radius $R∼N^{1/2}$, estimated from the two-dimensional planar disk area $\pi R^{2}$ divided by the cross-sectional area of a single bead, we expect ω∼1/R. Similarly, and with P=τ⋅ω, we expect P∼ωN∼R, a linear dependence on size, driving the need for larger μwheels that can perform more mechanical work or apply more power over a given amount of time. In addition to the available power, μwheels move at a velocity V∼ω⋅N^[20]^ leading to V∼R with larger μwheels translating faster (Figure 1B). Because both power and velocity are proportional to size, and while analogous nano-sized bots could be inhaled, they could not do significant work or be readily driven to desired sites once delivered. As opposed to approaches that use external fields to bias the impaction of inhaled nanoparticles,^[30,31]^ airborne transport of μbot building blocks for subsequent assembly overcomes these issues. Here, and to deliver μwheels, we first seed aerosol droplets with individual 4.5 μm beads for delivery into lung mimic airways. After delivery inside the airway, μwheels assemble in the aqueous film formed from the aerosol and are free to translate deeper into the lung (Fig 2A). Assembly of μwheels in situ has significant advantage as the building blocks are small enough to be aerosolized and delivered into lung pathways. Of additional note is that particles > 6 μm size^[32]^ are less susceptible to macrophage scavenging^[33]^ once delivered, further motivating the use of larger μbots. Because assembly is reversible, upon removal of the magnetic field, μwheels disassemble into individual beads for elimination by these natural mechanisms for dust and other foreign particles in the mucus lining.^[34]^ Additionally, these beads have been shown to have similar histological scores to alginate, a bioinert material commonly used in biomedical applications.^[35]^

To aerosolize the beads, a fluid aliquot containing beads is combined with an air stream inside a clinically and commercially available nebulizer, the flow rate of which determines droplet size distribution.^[24]^ We measure this distribution by directing aerosolized droplets into oil for subsequent imaging via optical microscopy (Figure 2B inset) where both droplet size and particle containing distributions are determined. Once aerosolized, we direct the droplets to surfaces where they impact, coalesce, and create a liquid film containing dispersed beads (Supplementary Video 2). The primary mechanisms of aerosol delivery are inertial impaction, gravitational sedimentation and Brownian diffusion.^[36]^ With these, larger particles > 5 μm tend to embed in the upper airway while smaller < 0.1 μm particles have the highest likelihood of making it deep within the respiratory tract. Though most are simply exhaled, such smaller particles can reach the lower bronchioles and alveolar lung regions where particle size would need to be 10 nm to reach via diffusion.^[34,37]^ While certainly aerosol-based drug delivery approaches use particle size in their targeting design, to do mechanical work or translate effectively once embedded, larger particles are required. Here the beads we use are available in the 1–5 μm range, overcoming the drawbacks of deposition in the upper airways by making available new mechanisms, including in situ assembly and rolling, to transport deeper into the lungs when desired.

As described previously and upon application of a weak rotating magnetic field, individual beads assemble into μwheels that use wet friction to move. To demonstrate that aerosolization does not negatively impact μwheel function, we compare velocities of μwheels composed of beads from solution to those assembled from aerosolized droplets (Figure 2C). In this, droplets are initially formed within an aerosolizer and condense on a surface in sufficient quantity to form a liquid layer. Within this layer and upon application of the magnetic field, beads assemble into μwheels with a velocity vs. size relationship (Figure 2C) similar to those assembled from solution. Small differences (Figure 2C inset) in radius distribution arise here due to local variation in bead concentration and resulting μwheel sizes during assembly.

**FIGURE 3:**
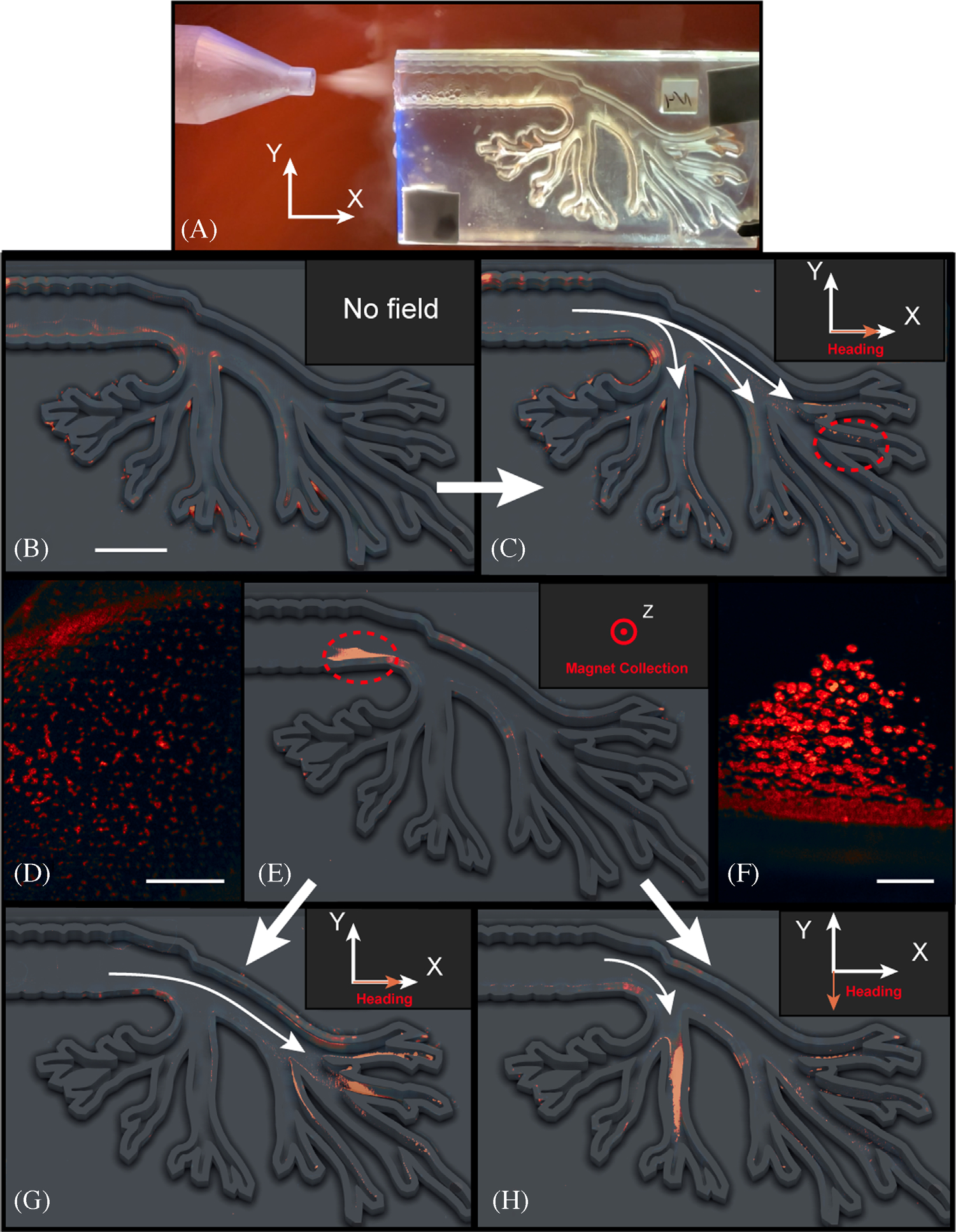
A, Aerosolization into 3D printed lung model. B, With illustration overlay, false color image of fluorescent, superparamagnetic beads dispersed throughout the model after aerosolization. Scale bar = 1 cm. C, Upon application of rotating magnetic field f=40Hz, B = 2.10 mT, axis of rotation $\overset{ˆ}{\Omega }=[0,cos(\pi /6),-1/2]$, μwheels form and D, travel down lung model pathways (Supplementary Video 3, scale bar = 1000 μm). Circled region in Fig 3C. E, For targeting, a permanent magnet can be used to capture aerosolized beads to form a bolus near the magnet. F, Upon magnet removal and with subsequent application of a weak rotating magnetic field, bolus μwheels (Supplementary Video 4, scale bar = 1000 μm) can be driven to desired branches. Circled region in Fig 3E. G, μWheels move deep into the right branches with identical applied field to Fig 3C. H, μWheels instead target the lower branches with a change of rotation axis $\hat{\Omega }=[cos(\pi /6),\;0,\;-1/2]$

For convenience, we investigate aerosolized delivery within a 3D-printed human pediatric-scale mimic, fabricated at a length scale to model transport from the bronchiole into the alveoli (Figure 3A). To aid imaging, we fluorescently label the beads and then aerosolize them within droplets sprayed into the model using a commercially available nebulizer, dispersing broadly throughout (Figure 3B). Upon application of the rotating magnetic field, μwheels subsequently assemble and roll down the bronchial tube to the lower bronchus in ~5–10 min (Figure 3C). Here, a rotating field is applied to drive μwheels in the +x direction to the ends of the channels where they accumulate. While such transport capability in general is useful for delivering μwheels deeper into the lungs, specific targeting may be useful in localized diseases. For example, to avoid systemic delivery of chemotherapeutic agents and the associated side effects, inhaled delivery of drugs for lung cancer could prove a promising approach. Progres0s here however has been limited due to concerns over toxicity and potential damage to healthy tissues throughout the rest of the lung.^[38]^ An approach where chemotherapeutic agents are delivered via μwheels to tumor surfaces could significantly enhance treatment and minimize side effects not only for the rest of the body but within the rest of the lung as well. To demonstrate targeting and with the purpose of creating a bolus, we place a magnet near the end of the model inlet where, upon aerosolization and entering into the model, beads collect (Figure 3E). Upon removal of the magnet and with application of the weak rotating magnetic field, μwheels form (Figure 3F) and can be directly driven to a desired endpoint (Figure 3G and Figure 3H). Note here that because of the relatively large size of the aerosolized building blocks, using fixed magnets for targeting deep in the lungs is not a workable strategy; in practice, magnets may not be required for targeting as beads can accumulate naturally at the upper end of larger scale systems due to their size.

One interesting aspect of aerosolized delivery is that, because of the high concentration of μwheels that this creates, swarming in the resulting assemblies can be observed. In other studies, we have shown that such swarms can be actuated and controlled differently, giving rise to net μwheel transport optimized for dispersal, or travel up inclines, or simply for speed. For the purposes of the measurements of Figure 3, the lung model was fixed horizontally as gravity plays an important role in μwheel transport, providing a load force and wet friction with adjacent surfaces. As one would expect, rolling downhill increases translational velocities while travel up steep slopes slows μwheel movement; however, we have recently shown that, with appropriate field application, both individual and swarms of μwheels can continue to move up inclines as high as 80°.^[39]^ We note here also that viscosity can play a significant role; with V∼ω for constant size μwheels, we expect V∼1/η and a slowing down as viscosity increases. For travel from the bronchiole to the alveoli over 10′s of cm, we expect μwheels to travel along the lower-viscosity sprayed fluid atop the higher-viscosity lung fluids already present while transport distances through the thicker mucus layer are significantly shorter and up to a few hundred μm.^[40]^ We have already demonstrated in previous studies the ability of these systems to deliver drug^[7]^ and the incorporation of lung dispersants^[41]^ to lower local viscosities is a potential strategy. Finally, and while we have chosen an approach with aerosolized droplets using a nebulizer for simplicity, we note that particle delivery could potentially be accomplished as a dry powder.^[42]^ Because the solid-phase building blocks are small enough and the particle size distribution well defined, once formulated, such an approach could provide advantages such as no need for propellants or more effective delivery for specific classes of drugs.

## 3 | CONCLUSIONS

Here we have demonstrated an in-situ μbot assembly approach that enables the delivery of μbots of size up to 80 μm and power up to 60 fW into the airways of a model lung. Our experimental results show the feasibility of aerosolizing building blocks by partitioning individual colloidal beads into droplets that are small enough to be delivered deep down pulmonary channels. With application of a weak rotating magnetic field, these individual particles assemble into large μwheels capable of rapid translation through a model pulmonary network.

## 4 | METHODS

### Magnetic Fields and Translation Studies:

To create and control the applied rotating magnetic field, we use a homebuilt actuation system with coils and signal generation software which generates a circular rotating field.^[43]^ The z axis consists of one 50 mm i.d. 400 turn coil below the sample while the x and y axes have two 50 mm i.d. 400 turn coils all incorporated in the microscope stage (Olympus OpenStand). The field strength was varied from 2.1 mT (Figure 2C and Figure 3) to 3.4 mT (Figure 2C) to demonstrate the flexibility of the approach. The field rotation frequency was kept constant at 40 Hz. The circular rotating field was cambered, or tilted, 30° from the z-axis for easier μwheel visualization. For initial translation studies (Figure 2C), the sample chamber consisted of two square 22 mm glass cover slips of 0.17 mm thickness sandwiched with a rectangular gasket cut from double-sided tape (RP32 VHB^™^ tape, 3 M, Maple, MN). To this, 4.5 μm diameter superparamagnetic beads (Dynabeads^®^ M-450 Epoxy, Thermo Fisher, density = 1.5 g cm^−3^) at an initial concentration of ~4•10^8^ beads ml^−1^ were diluted 200x with aqueous 0.2% sodium dodecyl sulfate (SDS) (Sigma-Aldrich) and added to the chamber. Videos were analyzed with custom open-source particle tracking software to measure rotation rates, radii, and velocities.^[44,45]^ Stuck beads and monomers were excluded, defined as those with velocity and diameter less than 5 μm s^−1^ and 6.75 μm, respectively.

### Bead–Laden Droplet Characterization:

100 μl of Dynabeads^®^ were fluorescently labeled by first adding 200 μl of aqueous 1 mg ml^−1^ rhodamine B solution and 700 μl of 0.2 wt% SDS aqueous solution. After 24 hr at room temperature, the solution was washed with 0.2 wt% SDS a total of 6x. Next, 100 μl of this solution was washed 3x with 0.1 wt% SDS and 5 vol% glycerine. The final solution was made after discarding the supernatant and adding 500 μl of 0.05 wt% SDS, 5 vol% glycerine, and 50 mg ml^−1^ of green food dye to increase the contrast and to form spherical droplets without air inclusions. The aerosol was created using the Pari LC^®^ Sprint Reusable Nebulizer (MMD 3.5 μm) with supply air at 3 lpm. For quantification of droplet size, the aerosol was sprayed over a thin layer of Type B immersion oil on a glass slide for 1 min. A brightfield macroscan of ~2 mm^2^ was taken with a 20x objective (Olympus IX81). This scan was performed using a stage loop where the camera and light source raster across a large area before being stitched together in software. Using threshold image analysis, the location and size of droplets and beads were determined. The data was then processed using a custom Matlab script to assign each bead to a specific droplet. Droplets below 0.5 μm in radius were not recorded due to image resolution limits.

### *Aerosolized* μ*Wheel Velocities*:

4 ml of Dynabeads^®^ diluted with 0.2% SDS aqueous solution to a final concentration of 4•10^6^ beads ml^−1^ was loaded into the nebulizer. The nebulizer was spaced 2 cm away and angled 45° toward a square 22 mm glass cover slip surrounded by a 5 mm high 3D printed retaining wall. The nebulizer was operated with a 3.5 lpm air supply until ~1 ml of solution was collected on the cover slip. The beads were then assembled into μwheels using the magnetic actuation system and microscope (Olympus OpenStand) with a field strength of 2.1 mT. For the control, 1 ml of the same solution was pipetted onto an identical cover slip with retaining wall, then actuated with the same field conditions. The μwheel velocities and radii were measured using previously mentioned tracking software.

### 3D Printed Lung Model and Targeting:

The 3D model was designed with a tracheal diameter of ~8 mm, corresponding to those measured for infants.^[46]^ The clear model was 3D printed (Form 3, FormLabs) and consisted of two halves which could be separated for viewing. A new model was printed for each experiment to avoid residual fluorescence staining. The model was first prepared by wetting with ~2 ml of 0.2% SDS solution. Next, fluorescently labeled Dynabeads^®^ were diluted to a concentration of 4•10^7^ beads ml^−1^ with 0.2% SDS solution. The nebulizer nozzle was placed at the entrance of the model while 1 ml of the diluted Dynabeads^®^ were aerosolized into the model with an air supply of 3.5 lpm for a total of 5 min. For experiments demonstrating targeting, a small permanent magnet was placed at the bottom of the model trachea, ~0.5 cm away from the first branch point. The measured field strength in the model at the point of collection was 130 mT.

For imaging, a macroscan using a TRITC filter (Olympus IX81) was taken after aerosolization to first characterize the initial distribution of beads throughout the model. For actuation, the device was placed on the microscope with magnetic actuation equipment (Olympus OpenStand). μWheels were assembled under an applied rotating field of 2.1 mT and actuated for 10 min for all experiments. The rolling direction of the μwheels was changed manually according to the targeted bronchial branch. Lastly, a second full macroscan of the device was performed to observe the movement of the fluorescently labeled beads after actuation.

## Supplementary Material

Supplementary Video 1

Supplementary Video 2

Supplementary Video 4

Supplementary Video 3
